# Distinct Alteration of Gene Expression Programs in the Small Intestine of Male and Female Mice in Response to Ablation of Intestinal Fabp Genes

**DOI:** 10.3390/genes11080943

**Published:** 2020-08-15

**Authors:** Yiheng Chen, Luis B. Agellon

**Affiliations:** School of Human Nutrition, McGill University, Ste. Anne de Bellevue, QC H9X 3V9, Canada; yiheng.chen@mail.mcgill.ca

**Keywords:** sex differences, transcriptome, fatty acid-binding proteins, Fabp, microarray analysis, nutrient metabolism, small intestine

## Abstract

Fatty acid-binding proteins (Fabps) make up a family of widely distributed cytoplasmic lipid-binding proteins. The small intestine contains three predominant Fabp species, Fabp1, Fabp2, and Fabp6. Our previous studies showed that *Fabp2* and *Fabp6* gene-disrupted mice exhibited sexually dimorphic phenotypes. In this study, we carried out a systematic comparative analysis of the small intestinal transcriptomes of 10 week-old wild-type (WT) and Fabp gene-disrupted male and female mice. We found that the small intestinal transcriptome of male and female mice showed key differences in the gene expression profiles that affect major biological processes. The deletion of specific Fabp genes induced unique and sex-specific changes in the gene expression program, although some differentially expressed genes in certain genotypes were common to both sexes. Functional annotation and interaction network analyses revealed that the number and type of affected pathways, as well as the sets of interacting nodes in each of the Fabp genotypes, are partitioned by sex. To our knowledge, this is the first time that sex differences were identified and categorized at the transcriptome level in mice lacking different intestinal Fabps. The distinctive transcriptome profiles of WT male and female small intestine may predetermine the nature of transcriptional reprogramming that manifests as sexually dimorphic responses to the ablation of intestinal Fabp genes.

## 1. Introduction

Fatty acid-binding proteins (Fabps) are highly abundant cytoplasmic proteins that are found in several mammalian tissues [1]. The function of these proteins are not fully known but they are thought to participate in the maintenance of intracellular lipid homeostasis [1,2]. The small intestine contains three types of Fabps, namely Fabp1 [3,4], Fabp2 [5], and Fabp6 [6,7].

Fabp1 (also known as L-FABP) was first found in the liver [4] but is also present throughout the small intestine with the highest abundance occurring in the proximal portion of the organ [8]. It can bind fatty acids with a preference for unsaturated long-chain fatty acids as well as an assortment of other hydrophobic molecules including bile acids and fibrates [9,10]. Studies have demonstrated a direct interaction between Fabp1 and peroxisome proliferator-activated receptors (PPAR), suggesting a role of Fabp1 in delivering PPAR ligands to the nucleus which in turn can lead to the expression modulation of PPAR target genes [11,12]. *Fabp1* gene ablation results in a variety of metabolic defects, including dysregulated hepatic lipid metabolism [13,14] and gallstone susceptibility [15]. A T94A variant of the human *FABP1* gene has been found to be associated with reduced body weight as well as altered glucose metabolic response to lipid challenge [16]. It can also influence the efficacy of lipid-lowering therapies [17]. Interestingly, the association between the T94A variant and increased fasting triacylglycerols and low-density lipoprotein (LDL)-cholesterol levels was only observed in females [18].

Fabp2 (also known as I-FABP) is restricted to the small intestine but distributed throughout the organ with its maximum abundance occurring after the midpoint of the organ [8]. Fabp2 shows a preference for saturated long chain fatty acids [9,10]. In vitro studies suggest that Fabp2 and Fabp1 enable the transfer of fatty acids from donor to acceptor membranes via different mechanisms: Fabp1 transfers fatty acids via an aqueous diffusion-mediated process whereas Fabp2 transfers fatty acids by direct collisional interaction with membranes [19]. The deletion of the Fabp2 gene in mice did not prevent dietary fat assimilation but influenced adiposity and glucose tolerance in a sex-dependent manner [20,21]. Studies on humans have found a potential association between the A54T human *FABP2* gene variant and insulin resistance, obesity as well as dyslipidemias in specific sexes in certain populations [22,23,24,25]. 

Fabp6 (also known as ILBP and I-BABP) is abundant in the distal region of the small intestine and displays a clear preference for bile acids although it is capable of binding fatty acids at lower affinity [8,9,26]. The targeted disruption of the *Fabp6* gene in mice demonstrated that Fabp6 is important in the intracellular transport of bile acids in enterocytes and the maintenance of the bile acid pool in the enterohepatic circulation [27]. Interestingly, Fabp6 is also found in the ovaries but not in the testes, and female mice lacking Fabp6 display a reduced ovulation rate compared to female wild-type (WT) mice [28]. Moreover, Fabp6 was found to be associated with the function of farnesoid X receptor (FXR) [29,30], suggesting a potential role of Fabp6 in modulating the activity of FXR in the nucleus. In humans, the T79M variant of human *FABP6* is associated with some protective effect on type 2 diabetes in obese subjects [31].

The targeted ablation of *Fabp1*, *Fabp2*, or *Fabp6* genes have all resulted in sexually dimorphic phenotypes [14,20,21,27,32]. Here, we compared the transcriptomes of small intestines from mice lacking either Fabp2 or Fabp6, or both of these Fabps to gain a comprehensive insight into the changes in the gene expression programs that occur in both sexes following the loss of these Fabps.

## 2. Materials and Methods 

### 2.1. Mice

Mice (*n* = 5 per cage) were housed in a temperature-controlled specific-pathogen-free facility and fed a standard lab diet (Purina 5001). *Fabp2*^–/^^–^ [21] and *Fabp6*^–/–^ [27] mice were maintained on the C57BL/6J background, which included one cross with a male C57BL/6J mouse, and interbred to generate the *Fabp2*^–/–^;*Fabp6*^–/–^ line. The use of mice was approved by the institutional animal welfare and policy committees in accordance with the policies of the Canadian Council on Animal Care (McGill University AUP5350; this project was initiated at the University of Alberta under Animal Protocol 270).

### 2.2. Preparation of RNA and Hybridization to DNA Microarray

The small intestine, starting from the base of the stomach and ending just before the cecum, was excised from fasted 10-week old mice littermates and then flushed with ice-cold saline prior to tissue homogenization. RNA was extracted from homogenates using Trizol (Invitrogen) and assessed for integrity (RIN > 7) prior to use in microarray analysis. The total RNA sample from 4 mice of each sex (male or female) and genotype (wild-type C57BL/6J, *Fabp2*^–/–^, *Fabp6*^–/–^, *Fabp2*^–/–^;*Fabp6*^–/–^) was analyzed as 2 biological replicates (RNA from 2 mice were pooled and sequenced as 1 sample). Fluorescently labeled cDNA probes generated from RNA samples were hybridized to Affymetrix 430A and 430B chips (total of 32, 16 per chip type) and processed as specified by the manufacturer.

### 2.3. Analysis of DNA Microarray Data

#### 2.3.1. Processing of Raw DNA Microarray Data

The microarray data were processed and analyzed using the R statistical environment and Bioconductor software [33] as illustrated in Supplementary Figure S1. Raw intensities from each gene chip were transformed to a log_2_ scale and quantile normalized using the Robust Multichip Average (RMA) algorithm with background correction [34]. The quality assessments were done by comparing the intensity between the biological replicates (See Supplementary Figure S2) and technical replicates (See Supplementary Figure S3). Boxplots were also used to evaluate the data quality across the chips (See Supplementary Figure S4). To improve the statistical power, the data were filtered based on their inter-quartile range and Entrez annotations [35]. The limFit function (limma package) [36] was applied to fit data into linear models to identify the genes with a differential expression between the male and female of the same genotypes and also between each of the Fabp gene-disrupted genotypes and wild-type mice of the same sex. The genes were screened with volcano plots (Supplementary Figure S5) generated using the EnhancedVolcano R package [37]. Genes with a false discovery rate <0.2 and an absolute log_2_ fold change > 0.5 were considered statistically significantly different. The Venn diagram plots were generated using the BioVenn website to present common and sex-specific differentially expressed (DE) genes induced by the deletion of Fabp genes [38]. See Supplementary Figure S1 for a more detailed description of the workflow and software resources used in the analyses.

#### 2.3.2. Gene Annotation and Functional Enrichment Analysis

The murine gene ID conversion and functional annotation of identified sex-biased genes and DE genes were done using DAVID Bioinformatics Resources (version 6.8) [39] with the default setting. The gene ontology (GO) terms in the biological process (BP) domains were extracted. The chromosomal locations of DE genes were determined from the Mouse Genome Database [40]. To display the metabolic patterns comprehensively, the relevant biological processes were grouped into four categories of interest in this study, namely lipid metabolism, carbohydrate metabolism, protein metabolism and sterol metabolism (complete lists are shown in Supplementary Table S2), and the number of unique DE genes in each category for each sex and genotype were counted and presented.

#### 2.3.3. Protein–Protein Interaction Network Analysis

In order to reveal sexually dimorphic protein interaction patterns, the corresponding proteins of the DE genes that were affected by the disruption of Fabp genes in each sex were used as seed proteins to identify the interacting proteins based on the literature-curated interactions in the NetworkAnalyst’s protein–protein interaction database (accessed on May 2020) [41]. Generic protein–protein interaction analysis was conducted using the International Molecular Exchange consortium (IMEx) interactome database using the default setting. The seed proteins were selected by the NetworkAnalyst algorithm based on the submitted gene lists. Subsequently, these seed proteins were used as the starting points to predict the functional protein network.

#### 2.3.4. Availability of DNA Microarray Data

The NCBI GEO accession number for the microarray data used in this study is GSE128862.

## 3. Results

### 3.1. Gene Expression Patterns in Male and Female Murine Small Intestine Are Distinct

**Figure 1:**
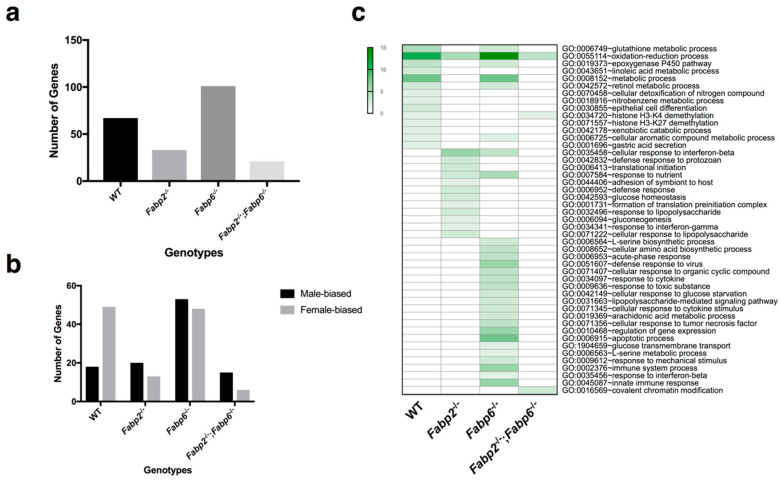
Sex-biased genes and functional annotation (gene ontology: biological process, GO: BP). (**a**) Comparison of the number of sex-biased genes identified in wild-type (WT), *Fabp2*^–/–^, *Fabp6*^–/–^, and *Fabp2*^–/–^;*Fabp6*^–/–^ mice. (**b**) The number of male- and female-biased genes in the small intestinal transcriptome across all genotypes. (**c**) Comparison of the biological processes enriched (*p*-value < 0.05) using sex-biased genes. The numbers of genes involved in each biological process are represented by the green color, darker shades indicate a greater number of genes. See Supplementary Table S1 for a complete list of sex-biased genes.

To address the difference in the gene expression in the small intestine between male and female mice with or without Fabps, transcriptome profiling was performed using microarrays in our study (see Supplementary Figure S1 for the description of the analysis workflow). Sex-biased genes were identified in all genotypes (Figure 1a). A total of 67 sex-biased genes were found in the WT mice. This number was reduced in the *Fabp2*^–/–^ and *Fabp2*^–/–^;*Fabp6*^–/–^ mice whereas the *Fabp6* gene deletion increased the number of sex-biased genes (Figure 1a), suggesting that the deletion of the *Fabp2* gene or both *Fabp2* and *Fabp6* genes made the intestinal gene expression of males and females more similar, while the disruption of the *Fabp6* gene increased the sexual dimorphism at the transcriptome level.

The genes that were identified as sex-biased were then further classified as either female-biased or male-biased. The results show that the number of female-biased genes, the sex-biased genes that have higher expression in females, is greater than male-biased ones in WT mice (Figure 1b), and this is consistent with previous findings [42,43]. The deletion of Fabp genes increased the proportion of male-biased genes, which might play roles in determining the male-specific phenotypes in Fabp gene-disrupted mice. In addition, most of the sex-biased genes reside on autosomal chromosomes (See Supplementary Figure S6).

The gene ontology-enriched analysis was applied to identify the biological pathways involving sex-biased genes. Many of the genes that show sexually dimorphic expression in WT small intestine are involved in nutrient and drug metabolism (Figure 1c), which is in accordance with other studies [44,45]. Some of the sexually dimorphic pathways in the small intestine that pre-existed in WT male and female mice were retained in mice lacking only one Fabp, i.e., either Fabp2 or Fabp6, whereas two pathways remained identifiable when mice were lacking both Fabps (Figure 1c). In general, the deletion of Fabp6 resulted in a greater number of sex dimorphic pathways compared to Fabp2, while the deletion of both Fabps resulted in the least number of sexually dimorphic pathways.

Thus, pre-existing differences in the gene expression program, as illustrated in the small intestinal transcriptome of WT male and female mice, may predetermine the intestinal gene expression program in response to the disruption of specific intestinal Fabp genes.

### 3.2. Genomic Responses to Ablation of Specific Fabp Genes Are Sexually Dimorphic

**Figure 2:**
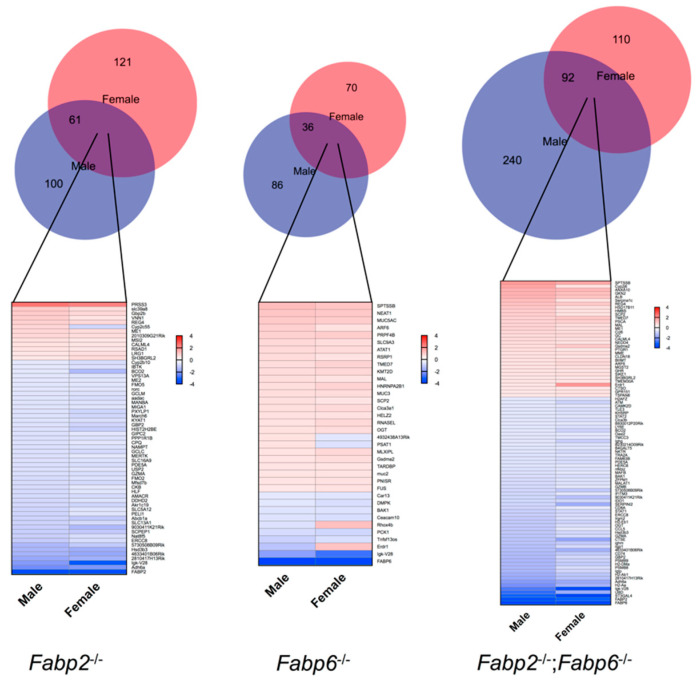
The comparison of the differentially expressed (DE) genes in different Fabp gene-disrupted male and female mice. The Venn Diagram of the common and sex-specific DE genes induced by the deletion of Fabp genes (top). The heatmap comparing the altered expression of the common DE genes shared by males and females (bottom). See Supplementary Table S1 for a complete list of DE genes.

We further evaluated the genomic responses to *Fabp2* and/or *Fabp6* gene disruption in male and female mice. DE genes were identified by comparing the transcript abundance between WT and Fabp gene-disrupted mice of each sex. In general, less than 50% of DE genes induced by Fabp gene ablations are shared by male and female mice (Figure 2, top, overlap of red and blue circles). Many of the shared DE genes (Figure 2, bottom, gene list) had distinct alterations in male and female mice. Some shared genes altered the gene expression in the opposite direction in males and females in response to the same Fabp gene disruption, such as *Cyp2c55* in *Fabp2*^–/–^ mice and *Psat1* in *Fabp6*^–/–^ mice. Notably, the numbers of DE genes identified in the small intestine of *Fabp2*^–/–^;*Fabp6*^–/–^ mice were much higher than the single Fabp gene-disrupted mice in the same sex. *Fabp2*^–/–^;*Fabp6*^–/–^ mice also have different sets and total number of DE genes compared to either single Fabp gene disruption regardless of the sex (See Supplementary Figure S7), implying a greater effect of the combined Fabp2 and Fabp6 deficiency on the overall gene expression program in the small intestine. Moreover, like sex-biased genes (See Supplementary Figure S6), most of the DE genes identified also reside on autosomal chromosomes (See Supplementary Figure S8).

To gain insight into the metabolic pathways differentially affected in males and females by Fabp gene ablations, gene ontology analysis using DE genes grouped by sex for each genotype was carried out. The top 10 biological processes having a *p*-value < 0.05 are shown in Table 1. Two groups of biological processes, namely metabolism-related and immune-related processes, comprise the major proportion of affected processes. Specifically, DE genes influenced by *Fabp2* or *Fabp6* gene single deletions are involved in metabolism-related biological processes, whereas combined *Fabp2* and *Fabp6* gene deletions mainly influenced immune-related biological processes. Furthermore, the males and females displayed distinct alterations of biological processes in response to the same Fabp gene ablation, which is concordant with previously reported changes observed in *Fabp2* gene-disrupted mice [46].

Together, the data show that Fabp gene deletions, either separately or combined, resulted in the sex-specific modification of gene expression in the small intestine. The identity of genes with altered expression in *Fabp2*^–/–^ and *Fabp6*^–/–^ mice are different from those in *Fabp2*^–/–^;*Fabp6*^–/–^ mice, suggesting that the biological processes that are affected when both *Fabp2* and *Fabp6* genes are missing are different from those when only one of these genes is absent.

### 3.3. Predicted Nutrient Metabolism Processes in the Small Intestine of Fabp Gene Ablated Mice Are Sex Biased

**Figure 3:**
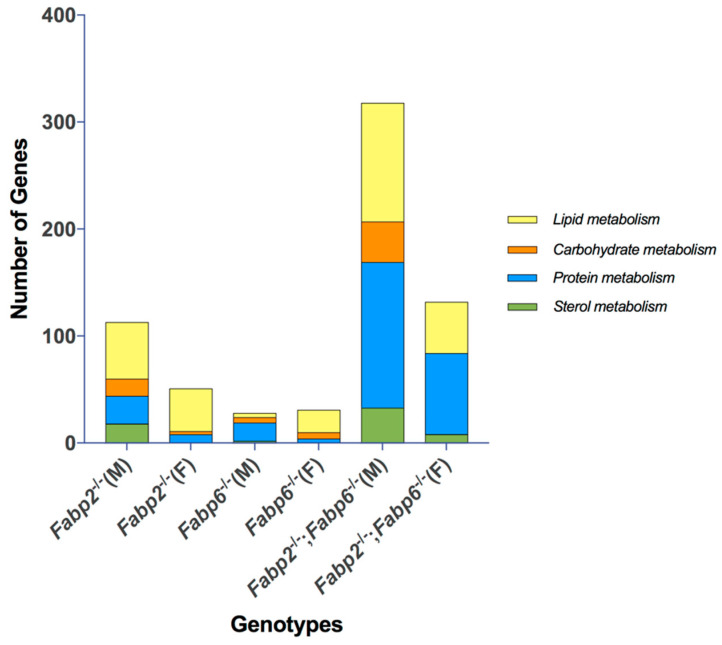
The comparison of the numbers of DE genes involved in key nutrient metabolism processes. The biological processes that comprise the nutrient metabolism categories are listed in the Supplementary Table S2.

Since the small intestine is the frontline for nutrient acquisition, transport, metabolism, as well as signaling, and where sex differences are also known to be pre-existing [47,48,49,50], we asked whether the genes involved in nutrient metabolism were influenced differently in male and female mice by the loss of specific Fabps. The affected biological process (GO:BP) identified was categorized according to macromolecule/macronutrient metabolism (carbohydrate, protein and lipid metabolism) and sterol/bile acid metabolism, and then the number of unique DE genes belonging to these categories was stratified according to genotype and sex. As shown in Figure 3, *Fabp2*^–/–^ and *Fabp2*^–/–^;*Fabp6*^–/–^ mice displayed similar metabolism patterns partitioned by sex. Specifically, all four categories of metabolism were affected more in males than in females, and the lipid and protein metabolism were influenced the most in both sexes. However, this pattern was not evident in the *Fabp6*^–/–^ mice, where the total numbers of DE genes involved in macronutrient metabolism were similar in male and female mice. In these mice, protein metabolism was influenced the most in males while lipid metabolism was greatly influenced in females. Surprisingly, only a few genes involved in sterol/bile acid metabolism were identified in both male and female *Fabp6*^–/–^ mice (Figure 3). For *Fabp2*^–/–^;*Fabp6*^–/–^ mice, there was a substantially higher number of DE genes involved in macronutrient and sterol metabolism compared to mice with single Fabp gene disruption.

**Figure 4:**
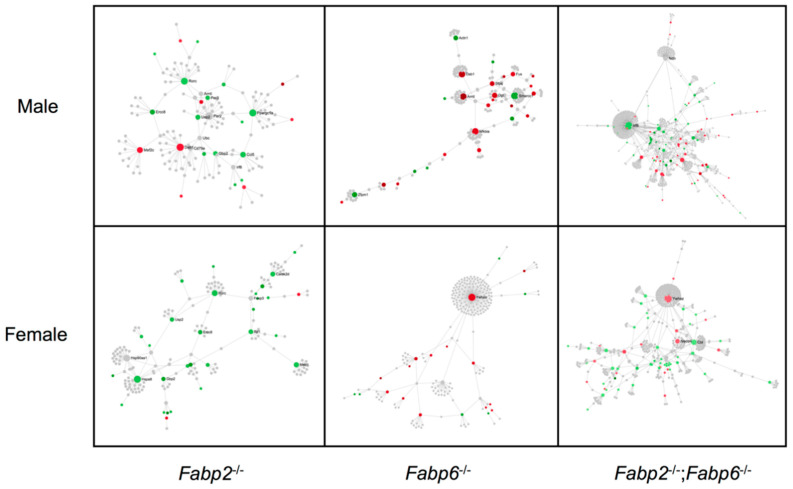
The protein–protein interaction networks of DE genes in different Fabp gene-disrupted genotypes (*Fabp2*^–/–^, *Fabp6*^–/–^, and *Fabp2*^–/–^;*Fabp6*^–/–^). Three types of proteins (nodes) are presented in the networks: seed proteins represented in green are encoded by downregulated genes and those represented in red are encoded by upregulated genes. Only the shortest-path first-order networks from the seed proteins are shown. Proteins represented in grey are known to directly interact with the seed proteins. The nodes are listed in the Supplementary Table S3.

We used the interaction network analysis to gain insight into the possible functions of DE genes based on the predicted interactions among their encoded proteins. Generally, males and females had very different network patterns in response to same Fabp gene ablation, suggesting sex-dependent biological responses, even though some nodes of the networks are shared (Figure 4). Specifically, the first-order networks of *Fabp6*^–/–^ and *Fabp2*^–/–^;*Fabp6*^–/–^ males contain more seed proteins, which are starting-point proteins extracted by the NetworkAnalyst to build networks, than females (28 for *Fabp6*^–/–^ and 90 for *Fabp2*^–/–^; *Fabp6*^–/–^ males; 24 for *Fabp6*^–/–^ and 66 for *Fabp2*^–/–^;*Fabp6*^–/–^ females) whereas the network of *Fabp2*^–/–^ males contains fewer seed proteins (25 for *Fabp2*^–/–^ males and 33 for *Fabp2*^–/–^ females) (Figure 4). When comparing the nodes that have more than one interacting protein, all mice (all genotypes, both sexes) have one node in common, forkhead box P3 (Foxp3), whereas only male mice share NF-KB (Nfkb1) and Sirtuin-1 (Sirt1). Interestingly, when both Fabp2 and Fabp6 are missing, Nfkb1, but not Sirt1, appear only in the network map of female mice. *Fabp2*^–/–^;*Fabp6*^–/–^ mice have a greater number of nodes in both sexes than the combined number of nodes in *Fabp2*^–/–^ and *Fabp6*^–/–^ mice, suggesting that a larger and more complex interaction network was affected when both Fabp2 and Fabp6 are missing.

## 4. Discussion

The existence of sexual dimorphism in lipid metabolism in the intestine has been described in both humans and mice [45,51,52]. As the most abundant cytoplasmic proteins that play pivotal roles in lipid metabolism in the small intestine, intestinal Fabps have been shown to exhibit sexually dimorphic expression in the small intestine of mice [20,27]. As for human intestinal FABPs [53], less is known about sex differences in the expression of their genes owing to the difficulty in obtaining samples for study. The targeted disruption of intestinal Fabp genes in mice also results in sexually dimorphic effects. The ablation of the *Fabp2* gene causes a much larger degree of metabolic disturbance in male *Fabp2*^–/–^ mice than female *Fabp2*^–/–^ mice [21,54]. Similarly, the ablation of the *Fabp6* gene induced differential alterations in male and female mice regarding bile acid metabolism [27]. The whole-body deficiency of the Fabp1 has also been shown to induce sexually dimorphic phenotypes [32,55] but since the *Fabp1* gene is expressed in many tissues, particularly in the liver, the metabolic consequences of intestine-specific deficiency of Fabp1 is not readily apparent from currently available *Fabp1*^–/–^ mouse models. It is clear from available studies that the ablation of genes encoding intestinal Fabps impacts on the expression of genes in a variety of tissues, in addition to the small intestine, and alter whole-body metabolism in a sex-dimorphic manner [27,54,55].

The sex dimorphic transcriptome is determined by many factors. Despite the males and females sharing a common autosomal genome, it is estimated that the sex-biased expression of genes in specific tissues ranges from less than 1% to 30%, depending on the sequencing techniques and statistical cut-offs used in the analyses [56,57]. Indeed, sex-dependent gene expression patterns are evident in the brain, liver, muscle, adipose tissue, and intestines of mice [43,58,59]. Sex differences in the transcriptome of a specific tissue are likely framed by the combined effects of biological sex as dictated by sex chromosomes, sex hormones or the sex-specific modification of the epigenome [60,61]. For example, substantial sex-biased gene expression is clearly evident in the small intestine of prepubescent mice and even as early as during embryo development [43,62]. In our study, nearly all the of DE genes detected in the intestinal transcriptome of all Fabp gene-disrupted genotypes were resident on autosomal chromosomes and very few were involved in sex hormonal-related processes. Moreover, protein–protein interaction analysis revealed that the major networks involving the DE genes in the majority of Fabp gene-disrupted genotypes included transcriptional signaling processes related to PPAR and FXR. It has been shown that PPAR and FXR themselves manifest a sexually dimorphic expression [63,64,65]. Given the fact that Fabps share the many ligands with these nuclear receptors, the loss of specific Fabps could further alter the ability of these transcription factors to regulate gene expression in a sex-dependent manner. All these findings might suggest the existence of sex-biased regulatory networks, which are constructed by proteins and RNAs with sex-biased abundance, including Fabps. Such networks, in turn, influence the internal availability of bioactive substrates such as nutrients and xenobiotics. Consequently, biological sex modifies the physiological responses to extrinsic interventions and differentiates the onset, development, and outcome of diseases in males and females [44,66].

This study provides insights into the potential biological roles and functional relationships of the intestinal Fabps. It was previously suggested that multiple Fabps in the small intestine might share some functions to ensure fatty acid and bile acid metabolism [20]. Fabp1 and Fabp2 show preference for binding fatty acids whereas Fabp6 prefers bile acids [9,26]. On the other hand, Fabp1 and Fabp6 can bind bile acids and fatty acids, respectively, at lower affinities [8,26]. Indeed, we found that mice lacking both Fabp2 and Fabp6 were viable. Interestingly, the network analysis revealed the loss of both Fabps affected a much larger number of processes in male mice than in female mice, similar to *Fabp2*^–/–^ mice. Future studies will uncover how these changes manifest at the organismal level. In addition, different regions of the small intestine have specialized metabolic and immune functions [67,68]. It would also be interesting to determine the nature of the changes in the gene expression program at these regions of the small intestine in males and females.

It should be noted that the findings of our analyses are applicable to a population of mice with the same age, fed the standard murine laboratory diet and housed under standard vivarium environmental conditions. Thus, our study does not inform on how the gene expression programs of male and female small intestine might evolve as a function of aging, in response to specific dietary challenges, changes in environmental conditions, nor does it provide information on changes in protein abundance or activity. Nevertheless, our findings provide a reference point for future studies to understand how biological sex impacts on the genomic responses associated with metabolic pathways involved in nutrient processing by the intestines, overall nutrient metabolism and susceptibility to various diet-induced metabolic diseases.

In conclusion, our study shows that sex is an important determinant of the intestinal transcriptome. Moreover, the pre-existing differences between males and females may govern the distinct alterations of the intestinal gene expression program manifested by males and females in response to the targeted inactivation of genes encoding the intestinal Fabps.

## Figures and Tables

**Table 1:** Gene ontology analysis for the DE genes induced in different genotypes. The top 10 biological processes that are enriched in each genotype are shown, as are the number of genes that fall within each process (count) and the *p*-values obtained.

*Fabp2*^–/–^ (M)	Count	*p*-Value	*Fabp2*^–/–^ (F)	Count	*p*-Value
GO:0055114~oxidation-reduction process	22	6.42 × 10^−8^	GO:0006749~glutathione metabolic process	5	6.03 × 10^−4^
GO:0006629~lipid metabolic process	17	6.02 × 10^−7^	GO:0006805~xenobiotic metabolic process	4	8.68 × 10^−4^
GO:0032922~circadian regulation of gene expression	7	7.89 × 10^−6^	GO:0008152~metabolic process	12	0.001148
GO:0008202~steroid metabolic process	7	4.98 × 10^−5^	GO:0042130~negative regulation of T cell proliferation	4	0.003863
GO:0048511~rhythmic process	8	6.51 × 10^−5^	GO:0035458~cellular response to interferon-β	4	0.004144
GO:0006631~fatty acid metabolic process	8	2.24 × 10^−4^	GO:0035729~cellular response to hepatocyte growth factor stimulus	3	0.00693
GO:0006805~xenobiotic metabolic process	4	8.33 × 10^−4^	GO:0055085~transmembrane transport	9	0.00846
GO:0006694~steroid biosynthetic process	5	0.001392	GO:0017144~drug metabolic process	3	0.008746
GO:0007623~circadian rhythm	6	0.001573	GO:0032922~circadian regulation of gene expression	4	0.012478
GO:0006641~triglyceride metabolic process	4	0.003454	GO:0006807~nitrogen compound metabolic process	3	0.012935
***Fabp6*^–/–^ (M)**			***Fabp6*^–/–^ (F)**		
GO:0045944~positive regulation of transcription from RNA polymerase II promoter	14	0.002734	GO:0006915~apoptotic process	10	0.001953
GO:0006814~sodium ion transport	5	0.004634	GO:0045779~negative regulation of bone resorption	3	0.002748
GO:0048511~rhythmic process	5	0.0051837	GO:0006397~mRNA processing	7	0.005133
GO:0006351~transcription, DNA-templated	20	0.005575	GO:0008652~cellular amino acid biosynthetic process	3	0.006677
GO:0033137~negative regulation of peptidyl-serine phosphorylation	3	0.008042	GO:0033137~negative regulation of peptidyl-serine phosphorylation	3	0.006677
GO:0008652~cellular amino acid biosynthetic process	3	0.008042	GO:0006094~gluconeogenesis	3	0.00721
GO:0051726~regulation of cell cycle	4	0.023003	GO:0061430~bone trabecula morphogenesis	2	0.014694
GO:0035020~regulation of Rac protein signal transduction	2	0.032085	GO:0051726~regulation of cell cycle	4	0.017872
GO:0006915~apoptotic process	8	0.03525	GO:0006564~L-serine biosynthetic process	2	0.019545
GO:0006810~transport	17	0.035583	GO:0045944~positive regulation of transcription from RNA polymerase II promoter	11	0.024183
***Fabp2*^–/–^;*Fabp6*^–/–^ (M)**			***Fabp2*^–/–^;*Fabp6*^–/–^ (F)**		
GO:0002376~immune system process	26	5.43 × 10^−9^	GO:0019886~antigen processing and presentation of exogenous peptide antigen via MHC class II	6	9.87 × 10^−8^
GO:0034341~response to interferon-γ	8	1.55 × 10^−7^	GO:0002376~immune system process	17	3.32 × 10^−7^
GO:0019882~antigen processing and presentation	10	2.21 × 10^−7^	GO:0019882~antigen processing and presentation	8	4.85 × 10^−7^
GO:0055114~oxidation-reduction process	32	2.85 × 10^−7^	GO:0034341~response to interferon-γ	6	2.97 × 10^−6^
GO:0035458~cellular response to interferon-β	9	2.95 × 10^−7^	GO:0035458~cellular response to interferon-β	6	3.04 × 10^−5^
GO:0042572~retinol metabolic process	7	2.00 × 10^−6^	GO:0006955~immune response	12	3.31 × 10^−5^
GO:0019886~antigen processing and presentation of exogenous peptide antigen via MHC class II	6	2.08 × 10^−6^	GO:0002504~antigen processing and presentation of peptide or polysaccharide antigen via MHC class II	4	5.60 × 10^−5^
GO:0006955~immune response	17	1.23 × 10^−5^	GO:0042130~negative regulation of T cell proliferation	5	4.31 × 10^−4^
GO:0007584~response to nutrient	9	2.63 × 10^−5^	GO:0060337~type I interferon signaling pathway	3	0.001154
GO:0006629~lipid metabolic process	22	2.64 × 10^−5^	GO:0031175~neuron projection development	7	0.00154

## Supplementary Materials

The following are available online at https://www.mdpi.com/2073-4425/11/8/943/s1. Supplementary Figure S1: Flowchart illustrating the microarray data analysis workflow. Supplementary Figure S2: Assessment of the quality of data by comparing signal intensity from the biological replicates using linear correlation (x axis, replicate 1 and y axis, replicate (2) after robust multichip average (RMA) processing. Supplementary Figure S3: Assessment of the quality of the data by comparing signal intensities of shared probes on Chip 430A (x axis) and Chip 430B (y axis) for each biological replicate using the linear correlation after a robust multichip average (RMA) processing. Supplementary Figure S4: Assessment of the quality of microarray data using boxplot after robust multichip average processing. Supplementary Figure S5. The volcano plots of differentially expressed genes in different Fabp gene-disrupted male and female mice. Supplementary Figure S6: Chromosomal location of sex-biased genes identified in wild-type (WT) and Fabp gene-disrupted mice. Supplementary Figure S7: Venn diagrams and heatmaps showing the number of differentially expressed genes in male and female intestinal transcriptomes of different Fabp gene-disrupted genotypes. Supplementary Figure S8: Chromosomal location of differentially expressed (DE) genes identified in male and female intestinal transcriptomes of different Fabp gene-disrupted genotypes. Supplementary Table S1: Complete lists of sex-biased genes and differentially expressed (DE) genes. Supplementary Table S2: List of the biological processes cited in Figure 3 categorized under lipid metabolism, carbohydrate metabolism, protein metabolism and sterol metabolism. Supplementary Table S3: List of nodes, edges and seeds identified in the networks shown in Figure 4.
