# Supplementary material for: Distinct Alteration of Gene Expression Programs in the Small Intestine of Male and Female Mice in Response to Ablation of Intestinal Fabp Genes
Source: Genes (Basel). 2020 Aug 15;11(8):943. doi: 10.3390/genes11080943 (PMC7465894; doi:10.3390/genes11080943)
Supplement: Supplementary file 1 [file Supplementary Figure S7.pdf › Supplementary Figure S7.pdf]

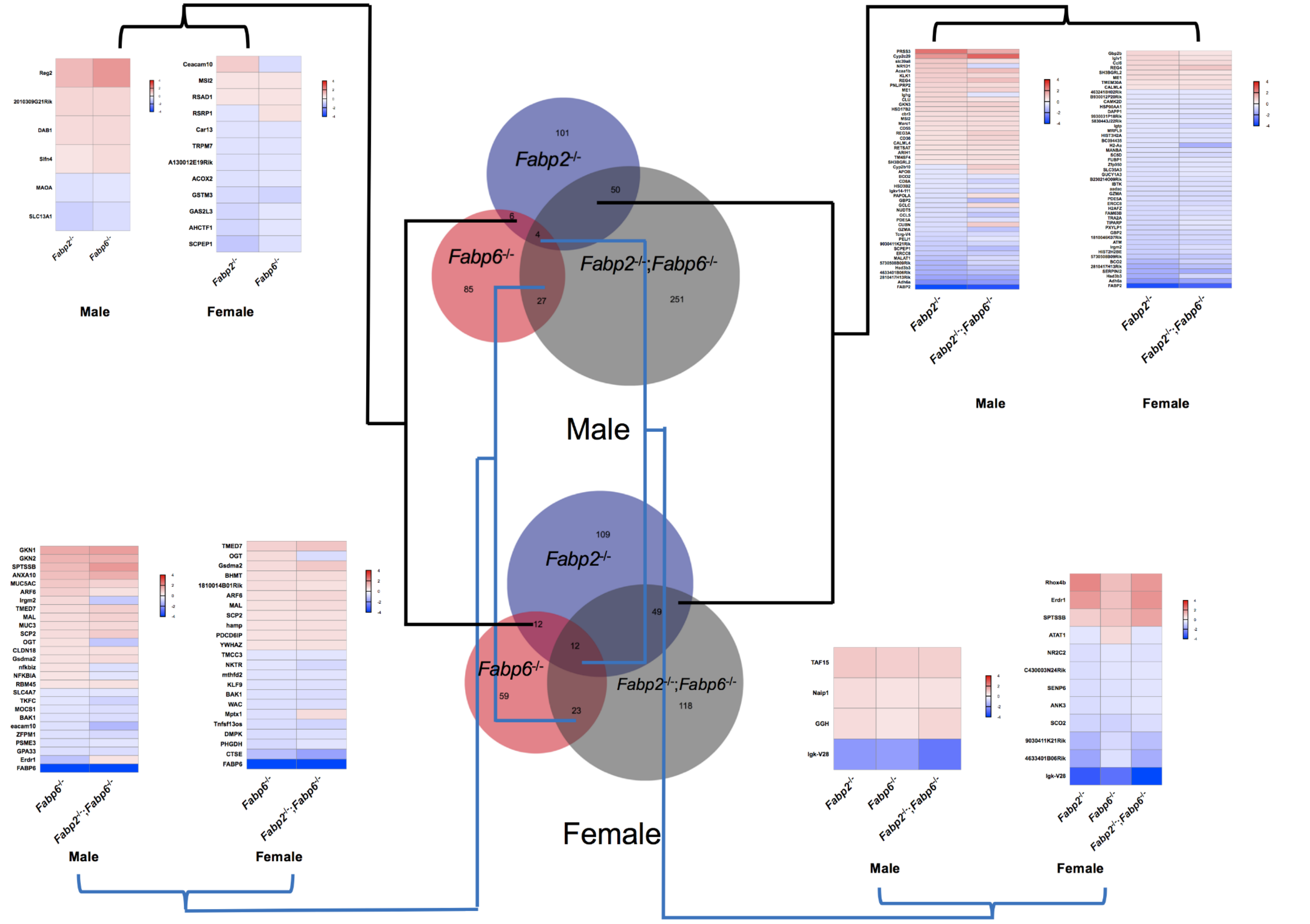

Supplementary Figure S7: Venn diagrams and heatmaps showing the number of differentially-expressed genes in male and female intestinal transcriptomes of the different Fabp gene-disrupted genotypes.
