# Supplementary material for: Distinct Alteration of Gene Expression Programs in the Small Intestine of Male and Female Mice in Response to Ablation of Intestinal Fabp Genes
Source: Genes (Basel). 2020 Aug 15;11(8):943. doi: 10.3390/genes11080943 (PMC7465894; doi:10.3390/genes11080943)
Supplement: Supplementary file 1 [file genes-11-00943-s001.zip › Supplementary Figure S1.pdf]

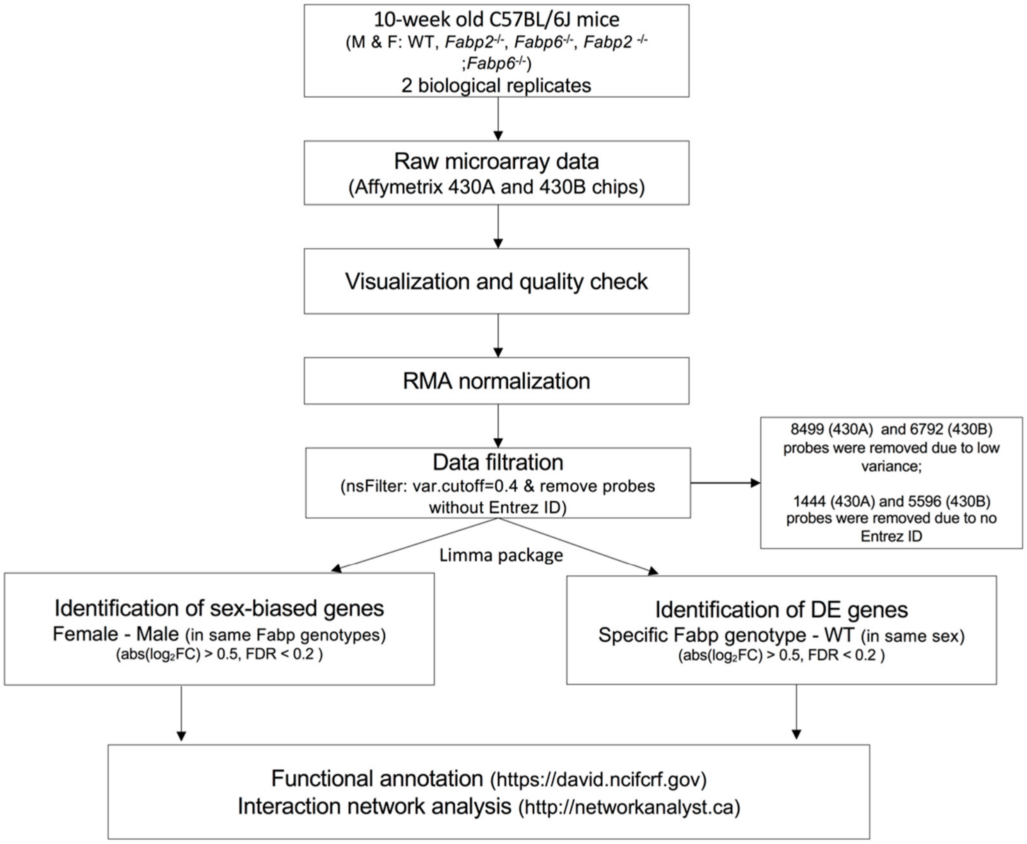

Supplementary Figure S1. Flowchart illustrating the microarray data analysis workflow. The raw intensity data were background corrected, $\log_2$ transformed and then quantile normalized using the RMA algorithm in the Affy package at the default setting. Quality of data was assessed by visualizing raw and processed data using scatter plot and boxplot. The data were then filtered using the nsFilter function (from genefilter package) according to two criteria: (1) with var.off=0.4 setting in nsFilter function, the least different 40% of genes across all genotype groups (genes have similar expression levels across all genotypes) were removed; (2) genes without entrez ID (i.e., genes without known biological information in National Center for Biotechnology Information database) were removed. The number of removed genes by each criterion are shown in flowchart (above). Next, two analyses were carried out: (1) one to capture changes resulting from disruption of Fabp genes in each sex (wild-type vs. gene-disrupted mouse for each sex; these are referred to as differentially expressed (DE) genes), and (2) another to capture differences between males and females of the same genotype (these are referred to as sex-biased genes). This step was performed using limFit function from the limma package with default setting. In brief, the limFit function fit multiple linear models (one per gene) to check the statistical significance of the observed difference for designated comparisons, either comparing males to females of the same genotype or comparing each genotype to wild-type of the same sex. The p-values were adjusted using Benjamini-Hochberg method to obtain the false discovery rates (FDR). The volcano plots were generated using the EnhancedVolcano R package and the genes with a false discovery rate <0.2 and an absolute $\log_2$ fold change (FC) >0.5 were considered statistically different.

Software packages and web-based computing resources used in the analyses

1. Affy package (https://bioconductor.org/packages/affy/ version 1.64.0)
2. Genefilter (https://bioconductor.org/packages/genefilter/ version 1.68.0)
3. Limma package (https://bioconductor.org/packages/limma/ version 3.42.2)
4. EnhancedVolcano package (https://bioconductor.org/packages/EnhancedVolcano/ version 1.4.0)
5. DAVID Bioinformatics Resources 6.8 (https://david.ncifcrf.gov May 2020)
6. NetworkAnalyst (https://www.networkanalyst.ca May 2020)
