# Supplementary material for: Distinct Alteration of Gene Expression Programs in the Small Intestine of Male and Female Mice in Response to Ablation of Intestinal Fabp Genes
Source: Genes (Basel). 2020 Aug 15;11(8):943. doi: 10.3390/genes11080943 (PMC7465894; doi:10.3390/genes11080943)
Supplement: Supplementary file 1 [file genes-11-00943-s001.zip › Supplementary Figure S2.pdf]

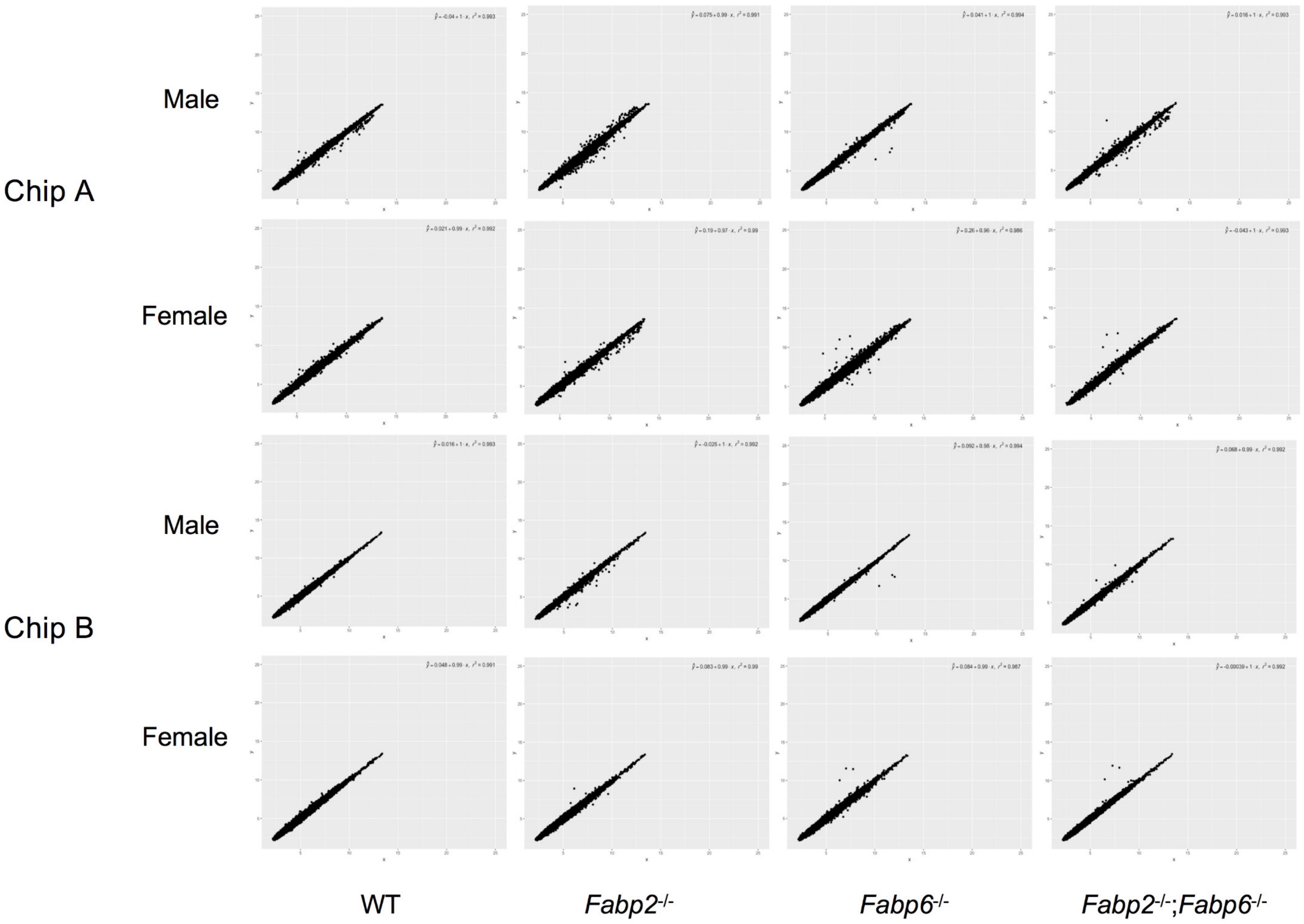

Supplementary Figure S2: Assessment of the quality of data by comparing signal intensity from the biological replicates using linear correlation (x-axis, replicate 1 and y-axis, replicate 2) after Robust Multichip Average (RMA) processing.
