# Supplementary material for: Distinct Alteration of Gene Expression Programs in the Small Intestine of Male and Female Mice in Response to Ablation of Intestinal Fabp Genes
Source: Genes (Basel). 2020 Aug 15;11(8):943. doi: 10.3390/genes11080943 (PMC7465894; doi:10.3390/genes11080943)
Supplement: Supplementary file 1 [file genes-11-00943-s001.zip › Supplementary Figure S5.pdf]

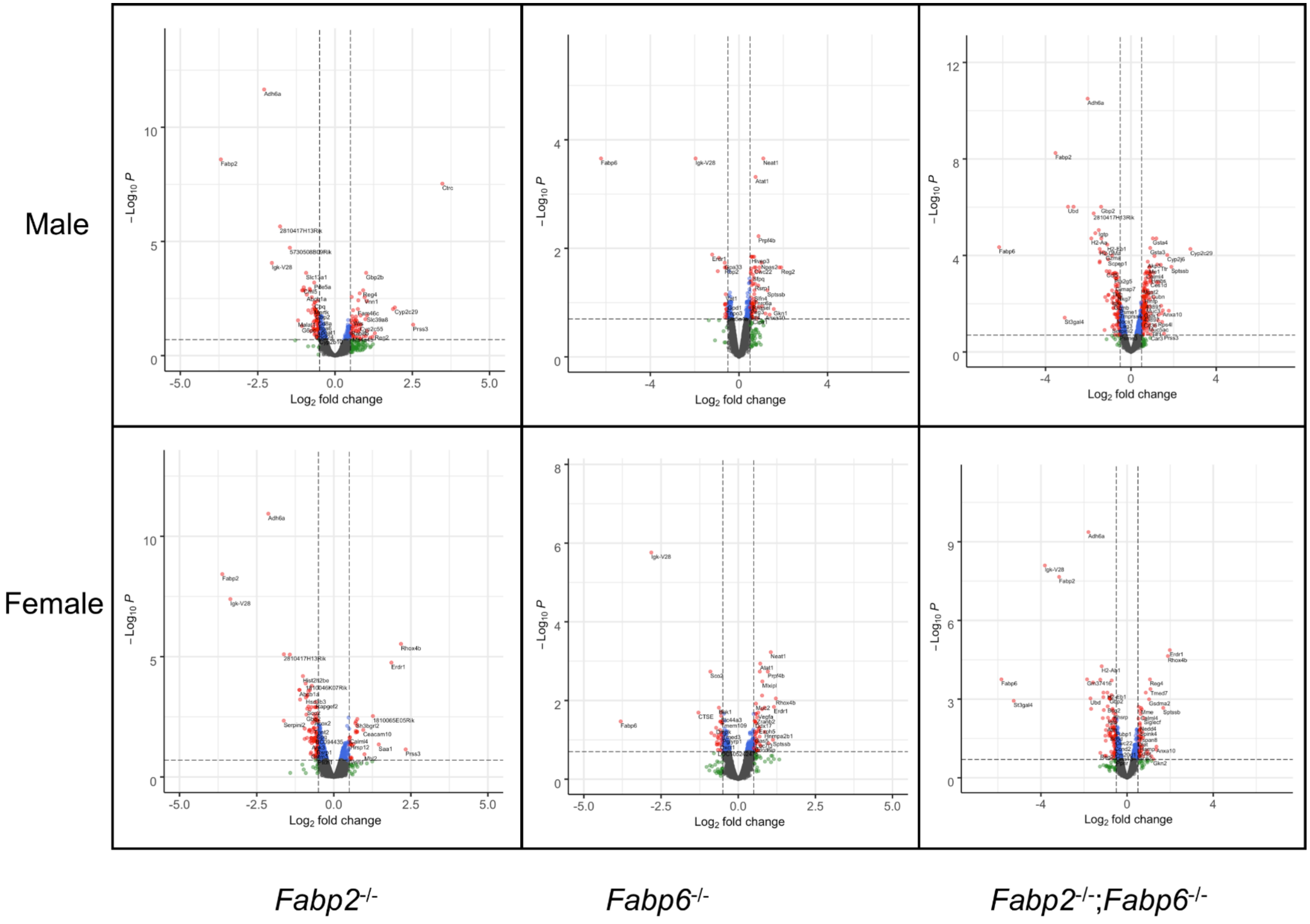


**Supplementary Figure S5. The volcano plots of differentially-expressed genes in different Fabp gene-disrupted male and female mice.** Filter 1: False Discovery Rate (FDR) < 0.2; Filter 2: absolute $\log_2$ fold-change > 0.5.
