# Supplementary material for: Distinct Alteration of Gene Expression Programs in the Small Intestine of Male and Female Mice in Response to Ablation of Intestinal Fabp Genes
Source: Genes (Basel). 2020 Aug 15;11(8):943. doi: 10.3390/genes11080943 (PMC7465894; doi:10.3390/genes11080943)
Supplement: Supplementary file 1 [file genes-11-00943-s001.zip › Supplementary Figure S8.pdf]

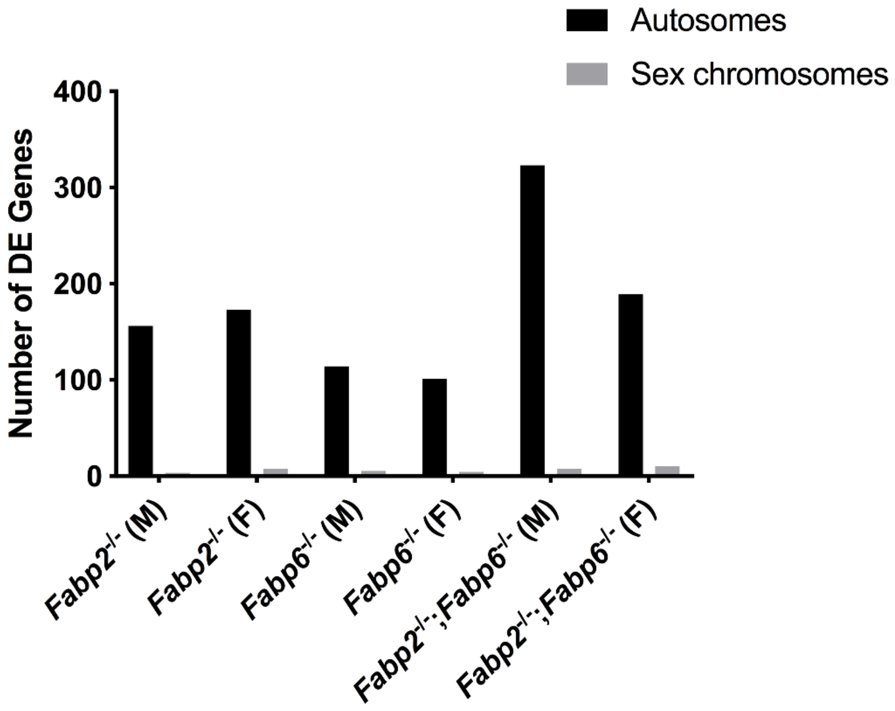

Supplementary Figure S8: Chromosomal location of differentially-expressed (DE) genes identified in male and female intestinal transcriptomes of different Fabp gene-disrupted mice.
